# Effects of Gender on Severity, Management and Outcome in Acute Biliary Pancreatitis

**DOI:** 10.1371/journal.pone.0057504

**Published:** 2013-02-28

**Authors:** Hsiu-Nien Shen, Wen-Ching Wang, Chin-Li Lu, Chung-Yi Li

**Affiliations:** 1 Department of Intensive Care Medicine, Chi Mei Medical Center, Tainan, Taiwan; 2 Department of General Surgery, Chi Mei Medical Center, Tainan, Taiwan; 3 Department of Medical Research, Chi Mei Medical Center, Tainan, Taiwan; 4 Department of Public Health, College of Medicine, National Cheng Kung University, Tainan, Taiwan; 5 Department of Public Health, China Medical University, Taichung, Taiwan; Klinikum rechts der Isar der TU München, Germany

## Abstract

**Background:**

We conducted a population-based cross-sectional study to examine gender differences in severity, management, and outcome among patients with acute biliary pancreatitis (ABP) because available data are insufficient and conflicting.

**Methods:**

We analyzed 13,110 patients (50.6% male) with first-attack ABP from Taiwan’s National Health Insurance Research Database between 2000 and 2009. The primary outcome was hospital mortality. Secondary outcomes included the development of severe ABP and the provision of treatment measures. Gender difference was assessed using multivariable analyses with generalized estimating equations models.

**Results:**

The odds of gastrointestinal bleeding (adjusted odds ratio [aOR] 1.44, 95% confidence interval [CI] 1.18–1.76) and local complication (aOR 1.38, 95% CI 1.05–1.82) were 44% and 38% higher in men than in women, respectively. Compared with women, men had 24% higher odds of receiving total parenteral nutrition (aOR 1.24, 95% CI 1.00–1.52), but had 18% and 41% lower odds of receiving cholecystectomy (aOR 0.82, 95% CI 0.72–0.93) and hemodialysis (aOR 0.59, 95% CI 0.42–0.83), respectively. Hospital mortality was higher in men than in women (1.8% *vs.* 1.1%, *p* = 0.001). After adjustment for potential confounders, men had 81% higher odds of in-hospital death than women (aOR 1.81, 95% CI 1.15–2.86). Among patients with severe ABP, hospital mortality was 11.0% and 7.5% in men and women (*p*<0.001), respectively. The adjusted odds of death remained higher in men than in women with severe ABP (aOR 1.72, 95% CI 1.10–2.68).

**Conclusions:**

Gender is an important determinant of outcome in patients with ABP and may affect their treatment measures.

## Introduction

Acute biliary pancreatitis (ABP) is a potentially fatal disease caused by gallstones and accounts for 35% to 70% of cases with pancreatitis worldwide [1], [2]. Several studies, however, reported a wider range, from <10% to >70% [3]. Although men are anatomically more susceptible to develop pancreatitis when gallstones are present [4], [5], ABP is usually more common in women. This fact is evidenced by the higher prevalence of gallstone disease in women in most countries [6]. Knowledge on effect of gender differences on the disease is important because it can help us understand the pathogenesis [4], [5], and serve as a guide for risk stratification [7]–[9], personalized medical care, and resource allocations [10], [11].

Studies on patients with other gallstone-related conditions [7]–[9] have indicated that gender differences occur in terms of disease severity, management and outcomes. Although these differences are theoretically observable in patients with ABP, the available data are insufficient and conflicting [2], [12]. For example, men were found to have more severe attacks of pancreatitis than women in one study (70% of 158 cases were ABP) [2]; however, this gender difference was not observed in another study [12]. Moreover, both studies are limited by a relatively small number of patients recruited from single institutions. Therefore, we conducted this national study to assess gender differences in patients with ABP. We hypothesized that men are more severe and have higher mortality than women with ABP, and that men receive fewer cholecystectomies during the same hospitalization period [13].

## Methods

### Ethics Statement

The review board of the Medical Research Committee in Chi Mei Medical Center approved the study (grant no. CMFHR10183) and waived the need for formal ethical approval and written informed consent from the participants because of the use of a reimbursement database containing encrypted and transformed data. Researchers using the database must sign an agreement based on the Computer-Processed Personal Data Protection Law and related regulations of the Bureau of National Health Insurance and the National Health Research Institute (NHRI) [14].

### Database

Patient data were retrieved from the Taiwan National Health Insurance Research Database. These data were released for research purposes by the NHRI [14]. The information in the inpatient database include sex, date of birth, encrypted patient identification number, residential or work area, dates of admission and discharge, medical institutions providing the services, the International Classification of Diseases, Ninth Revision, Clinical Modification (ICD-9-CM) codes of diagnoses (up to five) and procedures (up to five), outcome at hospital discharge (recovered, died or transferred), physician order codes and hospital charges.

### Definitions and Patients

ABP was defined by a principal diagnosis of acute pancreatitis (ICD-9-CM code 577.0) and a concurrent diagnosis of cholelithiasis (ICD-9-CM code 574.x) [15]. As described in previous studies [16]–[18], we included the following four severity criteria of acute pancreatitis based on the Atlanta classification scheme [19]: presence of intensive care unit (ICU) admission (as a surrogate of acute physiology and chronic health evaluation [APACHE] II score ≥8), organ failure, gastrointestinal bleeding (GIB), or local complication.

**Figure 1:**
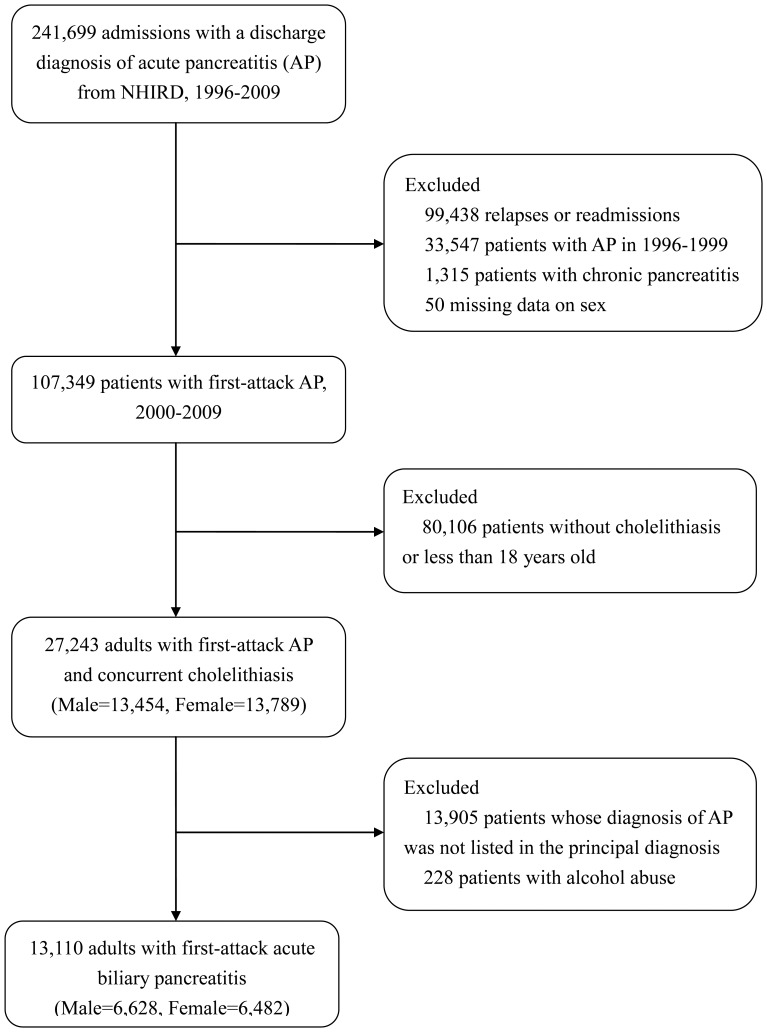
Study flow diagram. (NHIRD: National Health Insurance Research Database).

Figure 1 shows the enrollment process of patients with ABP, part of which has been described in a previous study [17]. Briefly, we initially identified patients with first-attack acute pancreatitis between 2000 and 2009, and then excluded those who were less than 18 years old or had no diagnosis of cholelithiasis. Finally, we excluded patients whose diagnosis of pancreatitis was not listed in the principal diagnosis or who had a concurrent diagnosis of alcohol abuse. The remaining 13,110 patients (50.6% male) were included in the analyses.

### Outcomes

The primary outcome was hospital mortality. Secondary outcomes included the development of severe ABP and the application of various treatment measures. The four severe criteria of ABP were examined jointly and separately. The management for ABP included endoscopic retrograde cholangiopancreatography (ERCP), cholecystectomy, and life-support measures (including total parenteral nutrition [TPN], hemodialysis and mechanical ventilation [MV]) [17].

### Covariates

Two groups of covariates were included in two sequential models (see below) to understand further the effect of gender on hospital mortality of ABP patients. First, baseline covariates that were used to characterize the patients and hospitals as well as the severity of pancreatitis included age (continuous variable), year of admission, urbanization (including urban, suburban and rural area), hospital level (including medical center [≥500 beds], regional [250–500 beds], and district hospitals [20–249 beds]), Charlson Comorbidity Index (0, 1, 2, 3+), imaging studies (including computed tomography [CT] and magnetic resonance imaging [MRI]), and the four severity criteria. Second, additional covariates that represent processes of care covered the length of hospital stay and various treatments (including ERCP, cholecystectomy and life-support measures). The Charlson Comorbidity index is a weighted summary measure of clinically important concomitant diseases adopted for use with ICD-9-CM coded administrative databases [20], [21].

### Statistics

Continuous variables are presented as median (inter-quartile range) because of a skewed distribution; whereas discrete variables are as counts or percentages. In the univariate analysis, we used the Mann-Whitney *U* test (for continuous variables) or the Chi-square test (for discrete variables) to compare differences between both genders. To account for hospital clustering, the effect of gender was analyzed using a logistic regression model with generalized estimating equations methods [22] and by specifying an exchangeable structure of a working correlation matrix to construct the regression models. The binary outcomes were regressed with a logit link function. Both univariable and multivariable analyses were performed to obtain the crude and adjusted odds. In the multivariable analysis of severe ABP risk, we only adjusted for the age, year of admission, urbanization, hospital level, Charlson Comorbidity Index (0, 1, 2, 3+), and imaging studies (CT/MRI). In the multivariable analysis of the odds of receiving various treatments, we adjusted the patient and hospital characteristics as well as pancreatitis severity (i.e., the aforementioned baseline covariates). Finally, in the multivariable analysis of mortality odds, we performed two sequential regression models by adjusting the two groups of covariates consecutively and additively. We also analyzed the effect of gender on hospital mortality enrolling only severe cases because almost all deaths occurred in patients with severe ABP.

Given that peptic ulcer is a known cause of GIB and that chronic renal disease is a known risk factor in performing hemodialysis, the two comorbid conditions were added as covariates when modeling such outcomes. Acute renal failure (ICD-9-CM code 584) was also added as a covariate when modeling the secondary outcome of hemodialysis because it is an important determinant. We assessed the potential problem of multicollinearity between covariates by examining the estimated slope coefficients and standard errors of the mean, and found no such indication.

We performed a sensitivity analysis in the multivariable logistic regression models by enrolling all ABP patients regardless of the coding position of acute pancreatitis to assess the robustness of the estimates. The data were analyzed using SPSS for Windows, version 17.0. (SPSS Inc., Illinois, U.S.A.). A two-tailed *p* value <0.05 was considered significant.

## Results

### Baseline Characteristics

Table 1 shows the characteristics of men and women with ABP. Compared with women, men were slightly younger, somewhat more likely to live in suburban areas, and had a higher Charlson’s score, which was reflected by the higher prevalence of most comorbid conditions, including respiratory diseases, hepatic diseases, peptic ulcers and cancers. However, the prevalence of renal diseases and diabetes mellitus was higher in women. Men also received more CT scans than women. MRI application and length of hospital stay were similar in both genders.

**Table 1:** Characteristics, imaging studies and length of hospital stay of patients with acute biliary pancreatitis by gender.

Variables^a^	Men (n = 6,628)	Women (n = 6,482)	P values
**Age, year**	62 (48–74)	64 (51–74)	<0.001
** ≥65**	45.2	49.1	<0.001
**Hospital level**			0.056
** Medical center**	33.3	34.8	
** Regional hospital**	46.2	46.1	
** District hospital**	20.5	19.0	
**Urbanization**			0.001
** Urban**	55.8	56.8	
** Suburban**	33.5	30.9	
** Rural**	10.7	12.3	
**Charlson Comorbid Index**			<0.001
** 0**	41.7	48.2	
** 1**	39.8	35.9	
** 2**	13.7	11.8	
** ≥3**	4.8	4.1	
**Comorbid conditions**			
** Cerebrovascular**	2.2	1.8	0.106
** Cardiovascular**	1.9	1.8	0.508
** Respiratory**	3.2	1.7	<0.001
** Renal**	2.4	3.1	0.012
** Hepatic**	26.4	20.7	<0.001
** Peptic ulcer**	19.2	15.6	<0.001
** Diabetes**	15.7	17.8	0.001
** Cancer**	2.7	2.1	0.025
** Others**	0.4	0.6	0.079
** Computed tomography**	42.4	39.2	<0.001
** Magnetic resonance imaging**	6.7	6.7	0.922
** Hospital length of stay, d**	6 (4–10)	6 (4–10)	0.562

a Values are expressed as median (interquartile range) or percentages.

### Outcomes

Table 2 shows the gender difference in the odds of severe ABP. When the four severity criteria were analyzed jointly, the odds of severe ABP were similar in both genders. However, when these severity criteria were analyzed separately, men had 44% higher odds of GIB and 38% higher odds of local complications. The odds of ICU admission or organ failure (≥1 system) were similar between men and women.

**Table 2:** The effect of male gender on the odds of severe attack in patients with acute biliary pancreatitis.

Severity criteria	Men (n = 6,628), %	Women (n = 6,482), %	Adjusted odds ratio (95%CI)^a^	P values
**Any**	15.7	14.2	1.08 (0.97–1.20)	0.133
**Intensive care unit admission**	7.7	7.1	1.09 (0.96–1.25)	0.163
**Organ failure**	8.0	7.9	0.94 (0.82–1.09)	0.454
**Gastrointestinal bleeding**	3.8	2.4	1.44 (1.18–1.76)^b^	<0.001
**Local complications**	1.7	1.2	1.38 (1.05–1.82)	0.017

a Multivariable logistic regression using Generalized Estimating Equations models adjusting for age, year of admission, urbanization, hospital level, Charlson Comorbidity Index (0, 1, 2, 3+) and imaging studies (i.e., computed tomography and magnetic resonance imaging).

b Peptic ulcer was forced into the model as an additional covariate because it is a cause of gastrointestinal bleeding.

Table 3 shows the gender differences in the delivery of various treatment measures. Men received more TPN (3.7% *vs.* 2.7%) and MV (3.7% *vs.* 3.1%), whereas women received more ERCP (18.7% *vs.* 17.2%), cholecystectomy 8.8% *vs.* 7.5%) and hemodialysis (2.4% *vs.* 1.6%). After the baseline covariates were adjusted, most of the above differences were still observed. Compared with women, men had 24% and 16% higher odds of receiving TPN and MV, respectively; however, men had 9%, 18% and 41% lower odds of receiving ERCP, cholecystectomy and hemodialysis, respectively, although the differences in usages of MV and ERCP were statistically insignificant.

**Table 3:** The effect of male gender on the management of patients with acute biliary pancreatitis.

Management	Men (n = 6,628), %	Women (n = 6,482), %	Adjusted odds ratio (95%CI)^a^	P values
**ERCP**	17.2	18.7	0.91 (0.80–1.03)	0.118
**Cholecystectomy**	7.5	8.8	0.82 (0.72–0.93)	0.002
**TPN**	3.7	2.7	1.24 (1.00–1.52)	0.046
**Hemodialysis**	1.6	2.4	0.59 (0.42–0.83)^b^	0.002
**MV**	3.7	3.1	1.16 (0.84–1.60)	0.368

ERCP: endoscopic retrograde cholangiopancreatography.

a Multivariable logistic regression using Generalized Estimating Equations models adjusting for age, year of admission, urbanization, hospital level, and Charlson Comorbidity Index (0, 1, 2, 3+), imaging studies (i.e., computed tomography and magnetic resonance imaging) and individual severity criteria (including intensive care unit admission, gastrointestinal bleeding, local complication and organ failure).

b Chronic renal disease and acute renal failure were forced into the model as additional covariates because they are risk factors of performing hemodialysis.

The risk of overall hospital mortality was 0.7% higher in men than in women (1.8% *vs.* 1.1%, *p* = 0.001) (Table 4). After adjustment for potential confounders (Model 2), men had 81% higher odds of in-hospital death than women. When only severe cases were enrolled, the risk of hospital mortality was 3.5% higher in men than in women (11.0% *vs.* 7.5%, *p*<0.001). The adjusted odds of death remained higher in men than in women with severe ABP (adjusted OR 1.72, 95% CI 1.10–2.68).

**Table 4:** Effects of male gender on outcomes in patients with acute biliary pancreatitis^a^.

Outcomes	Men (n = 6,628), %	Women (n = 6,482), %	Adjusted OR (95% CI)	
			Model 1	Model 2
**Hospital mortality**	1.8	1.1	1.86 (1.25–2.77)^b^	1.81 (1.15–2.86)^c^

OR: odds ratio; CI: confidence interval.

a Univariable and multivariable logistic regression models were performed with and without considering the cluster effect of hospitals using Generalized Estimating Equations models.

In model 1, adjusted covariates included age, year of admission, urbanization, hospital level, and Charlson Comorbidity Index (0, 1, 2, 3+), imaging studies (i.e., computed tomography and magnetic resonance imaging) and individual severity criteria (including intensive care unit admission, gastrointestinal bleeding, local complication and organ failure). In model 2, additional covariates included endoscopic retrograde cholangiopancreatography, cholecystectomy, total parenteral nutrition, hemodialysis and mechanical ventilation and length of hospital stay.

b *p* value  = 0.002.

c *p* value  = 0.010.

### Sensitivity Analyses

When all patients with ABP, regardless of coding position, were enrolled (total *n* = 27,015) (Figure 1), the gender differences in the odds of severity criteria of pancreatitis, treatments, and hospital mortality remained significant (except TPN), and the magnitude of the effect of gender was slightly attenuated for all except cholecystectomy. The male-to-female covariate-adjusted ORs were 1.37 (95% CI 1.19–1.59), 1.34 (95% CI 1.11–1.61), 1.12 (95% CI 0.95–1.33), 0.81 (95% CI 0.75–0.88), 0.64 (95% CI 0.52–0.80), and 1.56 (95% CI 1.20–2.02) for GIB, local complication, TPN, cholecystectomy, hemodialysis, and hospital mortality, respectively.

## Discussion

The results show that severity of and treatment for ABP differed between men and women. Men with ABP had higher odds of GIB or local complication, and received more TPNs but fewer cholecystectomies and dialysis therapies. Moreover, the covariate-adjusted odds of death was also higher in men, suggesting that unknown factors other than differences in care procedures may have contributed to their higher mortality.

Information on the subjects’ gender is seldom reported in studies of pancreatitis because it is not considered a risk factor of severe attacks or mortality [23], [24]. We speculated that the effect of gender may be obscured by the relatively low incidence of GIB and local complication in pancreatitis, as shown in our study. By analyzing the severity criteria separately, we were able to provide insight into the effect of gender on the disease process. The findings of increased odds of GIB and local complication in men with ABP may be supported by several observations from animal and human studies. For example, in animal models, the male sex has been found to be a risk factor of stress-induced gastric mucosal injury [25] and gastric ulcer [26]. In humans, men have a higher prevalence of peptic ulcer than women [27], [28] and older men have a higher risk of hospitalization for upper and lower GIB than older women [29]. The intensity of local inflammation is more extensive in men with symptomatic gallstone disease [30], which may contribute to men’s higher risk of local complications after laparoscopic cholecystectomy and the need of conversion to open surgery [9]. The aforementioned differences between men and women may be generally explained by sex hormone-mediated stress response and inflammatory reaction (i.e., sex bias) [31]. Nevertheless, further studies are still needed to explore other possible mechanisms involved in ABP.

The lower rate of cholecystectomy and the higher rate of TPN in men with ABP found in this study may be attributed to their higher rates of GIB and local complication. TPN may be needed for several patients with severe ABP who are intolerant for enteral nutrition; therefore, it is more likely to be provided for men with ABP. The frequency of cholecystectomy being performed during the same hospitalization period would be less in men with ABP because surgery for gallstones is usually postponed in severe cases to avoid complications [13]. The recent study of Nguyen and coworkers [15] investigated the effect of hospital volume on the rate of undergoing cholecystectomy or ERCP for ABP. Their results showed a less frequent provision of cholecystectomy in men and comparable uses of ERCP in both genders. Both of these results are in agreement with our findings. Although they failed to adjust for the disease severity of ABP, the magnitude of the effect of gender on the provision of cholecystectomy in their report (female-to-male OR 1.19) [15] is close to our results (male-to-female OR 0.82). These findings indicate that mechanisms other than disease severity (i.e., non-biological or gender bias) may exist in the differential delivery of cholecystectomy between men and women with ABP.

Hemodialysis was surprisingly delivered less to men than to women with ABP even after controlling for the status of kidney diseases. A higher prevalence of low estimated glomerular filtration rate (GFR) in women than in men has been shown to contribute to a higher rate of renal injury and mortality in female patients undergoing cardiac surgery [32]. Given that the data on the estimated GFR as well as on other laboratory and socio-clinical factors that may affect the decision in hemodialysis provision are not available, the causes of gender differences in the delivery of hemodialysis cannot be determined in this study.

In gallstone-related diseases, the male sex appears to be a risk factor of death. Similar to our finding on ABP, the risk of death is also twice higher in men than in women with symptomatic cholelithiasis [8]. Gender difference in mortality can only be demonstrated in studies with a large patient sample similar to the present study because of the low overall case fatality rate of ABP (2%–6%) [17], [33]. Furthermore, our study is strengthened by retrieving all treated patients from a national dataset, which can provide an unbiased selection and enhance its generalizability. The results of this study suggest that information on gender should be provided in future studies with regard to patients with ABP and that gender should be incorporated in the risk stratification for this particular disease.

Some limitations deserve comments. First, the definition of ABP in this study solely relies on the coding and not on the clinical criteria; thus, the accuracy cannot be verified. Moreover, the alcoholic cause of AP was under-coded in the database. We excluded patients who had “a concurrent diagnosis of alcohol abuse” instead of “a concurrent cause of alcohol abuse”. Second, our definition of severe ABP tended to include patients who experienced more severe attack and received intensive and/or invasive treatments. For example, several patients may not have been included if they had an APACHE II score ≥8 but were cared for outside the ICU, or if they had local complications but did not receive invasive procedures. Furthermore, several patients with organ failures may have been missed or under-coded because of the limited diagnosis space. However, the inclusion of patients who were miscoded as ABP or those who were more severe is likely non-differential, which tends to produce a bias in the observed effect toward the null. Third, several residual confounding factors may exist. For example, important clinical, laboratory and radiological features such as smoking, obesity, APACHE II score, and severity in CT scan are not available from the claims data. However, particular covariates that were enrolled may be used as surrogates for several unmeasured confounders. For instance, surrogate measures of ABP severity and the Charlson’s score, which incorporated several diseases associated with smoking, were already enrolled as covariates in the multivariable analysis. Therefore, the effect of residual confounding factors, although uncertain, is less likely to change the conclusion. Finally, we were unable to exclude patients whose ABP recurred more than four years after cholecystectomy. However, this limitation is also unlikely to reverse the observed gender difference in mortality because the incidence of recurrent ABP is considerably reduced after the definitive treatment for ABP [34].

In conclusion, our results demonstrate that gender is an important determinant of outcome in patients with ABP and show that gender affects some treatment measures for these patients. Further studies are needed to explore the underlying biological and/or non-biological mechanisms leading to the observed differences between men and women with ABP.
